# Lacrimal Gland, Ocular Surface, and Dry Eye

**DOI:** 10.1155/2016/7397694

**Published:** 2016-08-23

**Authors:** Chuanqing Ding, Edit Tóth-Molnár, Ningli Wang, Lei Zhou

**Affiliations:** ^1^Pharmacology & Pharmaceutical Sciences, USC Eye Institute, University of Southern California, Los Angeles, CA, USA; ^2^Pharmacology and Pharmacotherapy, Ophthalmology, University of Szeged, Szeged, Hungary; ^3^Ophthalmology, Beijing Tongren Hospital, Beijing Institute of Ophthalmology, Beijing, China; ^4^Singapore Eye Research Institute, Singapore; ^5^Department of Ophthalmology, Yong Loo Lin School of Medicine, National University of Singapore, Singapore; ^6^Ophthalmology and Visual Sciences Academic Clinical Research Program, Duke-NUS Medical School, National University of Singapore, Singapore

We hereby present to our readers what we have done in the past year, a special issue with 18 original research and review articles on lacrimal gland, ocular surface, and dry eye that covers wide topics in these areas.

As we have discussed in the call for papers, the main lacrimal gland, along with the ocular surface, which typically includes cornea, conjunctiva, meibomian gland, and other accessory glands, plays critical roles in the Ocular Surface System/Lacrimal Functional Unit. The interdependence and crosstalk among them are essential in maintaining the normal physiology and function of the ocular surface, providing essential nutrients, lubrication, and protection to the eyes to maintain their normal functions. Deficiencies in any of these tissues may lead to ocular surface diseases and vision impairment in its severe form.

There have seen enormous progress in our understanding of the components of the Ocular Surface System/Lacrimal Functional Unit since the new millennium. A quick PubMed search, using “Lacrimal Gland and Dry Eye” as key words, resulted in 1,459 publications tracing back to as early as 1946, with 919 of them published since 2000 (63% of the total). By using “Conjunctiva and Dry Eye” as key words, we found a total of 1,083 publications, with 740 of them published since 2000 (68% of the total; the earliest publication was in 1952). The main lacrimal gland is the major source of tear fluid, whereas in humans the conjunctiva occupies about 17 times more the surface area than the cornea. Increasing evidence suggests that both of them play essential roles in the etiology, progression, management, and prognosis of ocular surface diseases. The interactions among components of the Ocular Surface System/Lacrimal Functional Unit are vital to maintain the homeostasis of the ocular surface. Therefore it is critical to look at it as one unit in order to understand the pathogenesis of ocular surface diseases such as dry eye.

Dry eye is the most common reason for patient visits to the eye-care professionals, with epidemiology studies suggesting their prevalence ranging from 1-2% of the general population and can be as high as 30% in some groups, that is, seniors and women. Unfortunately, little is known about the etiology and pathogenesis of dry eye, and hence very limited management options are available at present. This further translates into enormous societal burden and economical losses, which has been estimated to be ~$55 billion/year in the United States alone, from this debilitating disease.

In this special issue, our authors have presented their findings and/or insights that span a wide range of topics, including the following: (i) novel polymer that may serve as potential eye drop excipient for treating dry eye; (ii) evaluation of the diagnostic value of McMonnies Questionnaire for dry eye screening in a multicenter study; (iii) exogenous hyaluronate playing a role in enhancing corneal epithelial cell wound healing; (iv) usefulness of measuring tear meniscus height and noninvasive keratograph tear breakup time as a simple and noninvasive screening test for dry eye patients; (v) high levels of 17*β*-estradiol being associated with increased matrix metalloproteinase-2 and metalloproteinase-9 activity in the tears from postmenopausal women with dry eye; (vi) meibomian gland dysfunction playing a critical role in dry eye in myopic teenagers; (vii) short-term effect of air pollution on occurrence of nonspecific conjunctivitis; (viii) a useful rabbit dry eye model for assessing conjunctival functionality; (ix) diabetes mellitus affecting the quality and stability of tear film in many different ways; (x) a study demonstrating that hemodialysis is able to effectively reduce tear osmolarity to normal values; (xi) safety and effectiveness of the intense pulsed light for treating meibomian gland dysfunction; (xii) a study on the influence of septal deviation on the prognosis of transcanalicular diode laser-assisted dacryocystorhinostomy; (xiii) role of silicon tube intubation in transcanalicular multidiode laser dacryocystorhinostomy; (xiv) topical diquafosol versus cyclosporine in dry eye patients following cataract surgery.

While these studies may only represent a small percentage of publications in these topics, they surely provide some exciting progress made in the past year. We hope these novel findings and insights presented by our authors will be of help to other investigators in their research areas and instill new ideas for our eventual goal of finding the causes of dry eye and effective therapies for this debilitating disease that inflicts millions of people worldwide.

*Chuanqing Ding* *Chuanqing Ding* *Edit Tóth-Molnár* *Edit Tóth-Molnár* *Ningli Wang* *Ningli Wang* *Lei Zhou* *Lei Zhou*

